# Topological phase transition in the antiferromagnetic topological insulator MnBi$_2$Te$_4$ from the point of view of axion-like state realization

**DOI:** 10.1038/s41598-023-42466-7

**Published:** 2023-09-28

**Authors:** A. M. Shikin, T. P. Estyunina, A. V. Eryzhenkov, N. L. Zaitsev, A. V. Tarasov

**Affiliations:** 1 St. Petersburg State University, St. Petersburg, Russia 198504; 2 Institute of Molecule and Crystal Physics, Subdivision of the Ufa Federal Research Centre of the Russian Academy of Sciences, Ufa, Russia 450075

**Keywords:** Phase transitions and critical phenomena, Electronic structure

## Abstract

This work aims to study the conditions of topological phase transition (TPT) between the topological and trivial states in the antiferromagnetic topological insulator (AFM TI) MnBi$_2$Te$_4$ and propose some theory about the relationship of this TPT with the possibility of axion-like state realization in this material. Using the density functional approach we have analyzed the changes in the electronic and spin structure of topological surface states (TSSs) and the nearest conduction and valence bands (CB and VB) including the changes in the bulk band gap as well as the Dirac point (DP) gap in TSSs under variation of the spin-orbit coupling strength in the region of the TPT for infinite crystal and slab with a surface both. We have shown that in both cases the TPT occurs with inversion of the contributions of the Bi-$p_z$ and Te-$p_z$ states of different parity at the gap edges related to change in the gap sign. In the case of surface calculations, the Bi-$p_z$ and Te-$p_z$ states at the edges of the bulk band gap and their inversion at the TPT point are transformed into the TSSs with an energy gap at the DP. In this case the TPT takes place without closing the band gap, i.e. with a “jump” through zero and the formation of the nonzero gap during such a transition. Our calculations show that the TPT point is also characterized by an inversion of the out-of-plane spin polarization $s_z$ at the $\Gamma$ point for lower and upper parts of the Dirac cone and a significant spatial redistribution of the TSSs between the surface and the bulk. We suppose that the nonzero Dirac gap can have some relationship with the formation of the axion-like state, which presumably couples nonmagnetic spin-orbit and magnetic contributions at the boundary between the topological and trivial phases for a system with parameters close to the TPT conditions. A practically realized system is proposed - the AFM TI with a stoichiometry close to that of MnBi$_2$Te$_2$Se$_2$ with partial (about 50%) substitution of Te atoms for Se atoms in MnBi$_2$Te$_4$ which can be an experimental platform for the implementation and experimental analysis of the TPT and the corresponding possibility of the axion-like state realization in Condensed Matter. Besides, such system could serve as a good platform for studying the dynamic axion effect, where the axion field fluctuations are maximised when a small external field is applied to the system which state is close to the TPT.

## Introduction

The idea of the axion as a hypothetical particle, first proposed in quantum chromodynamcs^1–4^, is now already extended in Condensed Matter to describe such exotic phenomena as the topological quantized topological magnetoelectric (ME) and quantum Hall effects (including the zero-plateau ones), realization of the topological axion insulator state, static and dynamic axion response of topological systems (including axionic polariton), axion instability, etc.^5–22^. Axion was first postulated as a hypothetical particle to solve the charge-parity problem in the interaction between quarks in particle physics, causing the introduction of an additional term into the Lagrangian of the system, determined by a pseudoscalar field $\theta$, the magnitude of which can vary depending on the type of the system symmetry^1–4,7,8^. On the other hand, currently the axion (*a*) is considered, in particular, as a quasi-particle formed as a result of the interaction in fermion-antifermion pair (*f* and $f^*$, for fermions of opposite chirality) like $f+f^*\rightarrow a$^23^ or two photons ($\gamma$ and $\gamma ^*$, an ordinary and a virtual (dark) photon), as representatives of massless fermions $\gamma +\gamma ^*\rightarrow a$, with violation of the time reversal and parity symmetry, which is accompanied by the opening of an energy gap in the formed dispersion (that determines the mass of the axion) during such an interaction^3,4,23^. At present, based on a similar representation, the idea of the axion is also used in condensed matter theory, cosmology and astrophysics, in superstring theory, and is also considered as a possible component in the interaction with dark matter^3,4,23,24^. At the same time, in many axion models, to describe the axion field, the complex field $\mathrm {\Phi }=\chi e^{i\theta }$ is introduced, which gains nonzero vacuum expectation value dependent on chiral symmetry of the system. In Condensed Matter Physics (first of all for magnetic TIs)^2,5–22,25–27^ the axion term was taken from the topological field theory to describe the ME response (static and dynamic) in topological medium to an external electromagnetic excitation through the axion action $S_{\theta }=\frac{\theta }{2\pi }\frac{e^2}{2\pi \hbar c} \int d^3xdt {\textbf {E}} \cdot {\textbf {B}}$ due to the similarity of the expression for the electrodynamic response for a hypothetical axion particle introduced to solve the charge conjugation parity (CP) symmetry violation problem in particle physics^1,5–8,26^. Here $\frac{\theta }{2\pi }$ is the axion term, or axion angle in the representation of the wave function in systems with different types of symmetry (playing the role of a pseudoscalar field), the value of which changes under the transition between a topological and a trivial medium with different types of symmetry, $\frac{e^2}{\hbar c}=\alpha$ is the fine structure constant, *E* and *B* are the electric and magnetic fields. In the presence of time-reversal symmetry $\theta$ takes a quantized value $\theta =\pi$ for topological media, while $\theta =0$ in trivial media (see, for instance^5–8^). At the boundary between the topological and trivial media $\theta$ changes from $\pi$ in TI to 0 in vacuum (as a trivial insulator) across the surface.

On the basis of this correlation, the concept of an axion insulator was introduced in Condensed Matter Physics, as a magnetic TI structure with the opposite out-of-plane spin orientation at opposite surfaces, characterized by the quantized ME response proportional to $\frac{1}{2} \frac{e^2}{\hbar c}$ and the corresponding quantized value of the surface conductivity, which is determined by the quantized $\frac{\theta }{2\pi }$ value of the axion term (by $\pi$) in electromagnetic Lagrangian of 3D TI^7–19^. In this case, it is precisely a change in $\theta$ by $\pi$ at the boundary between the topological and trivial medium (i.e. at the $\theta$-boundary) provides the realization of the topological quantized ME and half QH effect at the given $\theta$-boundary, i.e. on the surface of such an axion insulator.

Initially, the idea of an axion insulator in Condensed Matter was developed in refs^16–19^, where the axion insulator was created on the basis of a layered topological structure, in which the layers of magnetically doped TIs (Cr- and V-doped TI (Bi,Sb)$_2$Te$_3$) with different coercive force and opposite orientation of magnetic moments interacted through a layer of nonmagnetic TI. It is for the axion insulator that the zero-plateau quantum anomalous Hall effect (ZPQAHE) is realized and the topological ME effect is assumed. For the implementation of the last at the boundary between the topological and trivial medium (or on the surface of the magnetic TI) it is necessary to introduce the axion term presented above into the Lagrangian, when changing the value of $\theta$ from $\pi$ to 0^5–8^. At the same time, recently a new type of intrinsic magnetically ordered AFM TI MnBi$_2$Te$_4$ with a natural opposite orientation of magnetic moments in neighboring magnetic layers has been synthesized^28–32^, which can be a good promising platform, where representation of axion insulator can be reached (and investigated) in a more natural and optimal way. AFM TI MnBi$_2$Te$_4$ is a layered compound consisting of the septuple layer (SL) blocks (Te-Bi-Te-Mn-Te-Bi-Te) separated by van der Waals (vdW) intervals^28–32^. In this case, the Mn atoms arranged in one plane inside each SL are coupled by a ferromagnetic (FM) interaction, while coupling between magnetic Mn atoms in the neighboring SLs has an AFM character^28–32^. It was shown that this magnetic TIs can indeed be axion insulators with opposite orientation of magnetic moments at opposite surfaces (under condition of an even number of magnetic SLs) and realization of the ZPQAHE. In this AFM TI each of the surfaces is characterized by a contribution to the conductivity equal to half the conduction quantum, but with the opposite sign $\pm \frac{1}{2}\frac{e^2}{h}$, thereby compensating each other, with the possibility of displaying a topological ME response.

It should be noted here that the idea of the axion was originally introduced and investigated in Condensed Matter Physics under analysis of the possibility of the manifestation of the dynamic axion effect in magnetic TIs^10–15^, i.e. excitations of a dynamic axion field under the influence of electromagnetic radiation (in the presence of a magnetic field), which can be registered experimentally by generating an axion polariton^10^. Wherein, the concept of the static ($\theta _0$) and dynamic ($\delta \theta$) parts of the axion term (axion field) was introduced: $\theta (r,t)=\theta _0+\delta \theta (r,t)$. The static part can be considered as a dimensionless pseudoscalar parameter (or pseudoscalar axion field). The dynamic part is determined by the change in $\theta$ created by an external action (by external electromagnetic field in combination with an applied magnetic field, breaking the time reversal symmetry and leading to modulation of the value $\theta$ with respect to $\theta _0=\pi$)^5,8,10–15^. In this case, it was shown that for the effective implementation of the dynamic axion effect (in addition to the generation of the time-dependent AFM fluctuations), the conditions for the minimum size of the nonmagnetic band gap formed primarily by the spin-orbit coupling strength ($\lambda _\text {SOC}$) must be satisfied^8,10–14,22^. It should be noted here that the region of the band gap minimum corresponds to the topological phase transition (TPT) from a topological to a trivial insulator^5–8,31^, when the band gap value should pass through zero with the corresponding inversion of states at the band gap edges. In addition, as noted earlier, the region of the TPT corresponds to the change of the axion term $\theta$ from $\pi$ to 0 under variation of $\lambda _\text {SOC}$ and effective formation of a dynamic axion in the solid. This means that it is precisely the system with the effective SOC value corresponding to the TPT from the topological to the trivial state (with changing $\theta =\pi \rightarrow 0$) and the minimum size of the formed gap that should correspond to the greatest extent to formation of the axion-like state, which is presumably localized at the $\theta$-boundary between the crystal and vacuum. At the same time, we assume that the axion realized in the region of the TPT affects the structure of the TSSs (including corresponding variation of the energy gap at the Dirac point (DP), and the analysis of changes in their electronic and spin structures, as well as the redistribution of the TSSs under the TPT, can shed light on the problem and conditions of the realization of an axion-like state in magnetic TI.

In previous publications devoted to the study of the electronic structure of MnBi$_2$Te$_4$ and the variation of the gap opened at the DP, it was shown that for different samples of this TI, the size of the Dirac gap can vary over a wide range: from values of 80-90 meV, based on theoretical estimates^28–35^ or 50-60 meV, according to the experimental studies^33–37^, and up to several meV, and lower down to gapless-like dispersions^35–40^. At the same time, it is well known that if one theoretically changes (reduces) the value of $\lambda _\text {SOC}$ in MnBi$_2$Te$_4$, then this will lead to a significant change (decrease) in the size of the bulk Dirac gap in such a model system^30,31,41^, up to the re-inversion states at the boundaries of the Dirac gap and to the TPT of the system from the topological to the trivial state. Similar changes (as we show below) also take place for the energy gap with variation of $\lambda _\text {SOC}$ for other types of the intrinsic magnetic TI^12–14^. And what is important, the Dirac gap minimum exactly corresponds to the region of the TPT, where the axion-like state should manifest itself to the greatest extent. In addition, it is also known that the energy gap can be modulated by introducing Pb and Sn atoms at Mn positions in Mn$_{1-x}$Pb$_x$Bi$_2$Te$_4$^42^ and Mn$_{1-x}$Sn$_x$Bi$_2$Te$_4$^43^ systems. In this case it is assumed that at sufficient concentrations of impurity it is possible to realize TPT through a gapless state from a topological to a trivial insulator, too.

This work is devoted to studying the changes in the bulk and surface electronic structure of MnBi$_2$Te$_4$ under TPT between the topological and trivial phases from the point of view of the possibility of realization of the axion-like state in AFM TI, which, as we assume, can be considered as a quasiparticle that determines the interaction between the topological and trivial phases at the boundary between them. The analysis is carried out on the basis of the electronic structure calculations provided by the DFT method with modulating $\lambda _\text {SOC}$ in the region of values nearby the TPT, at which the energy gap at the DP reaches minimum and the gap changes its sign during the transition of the system from the topological to the trivial state. In this regard, we focus on the study of the conditions for the formation of the energy gap minimum at the DP, as well as the analysis of parameters that can affect the Dirac gap in the region of its minimum value, when $\lambda _\text {SOC}$ is modified.

### Modification of the bulk electronic structure upon topological phase transition

**Figure 1:**
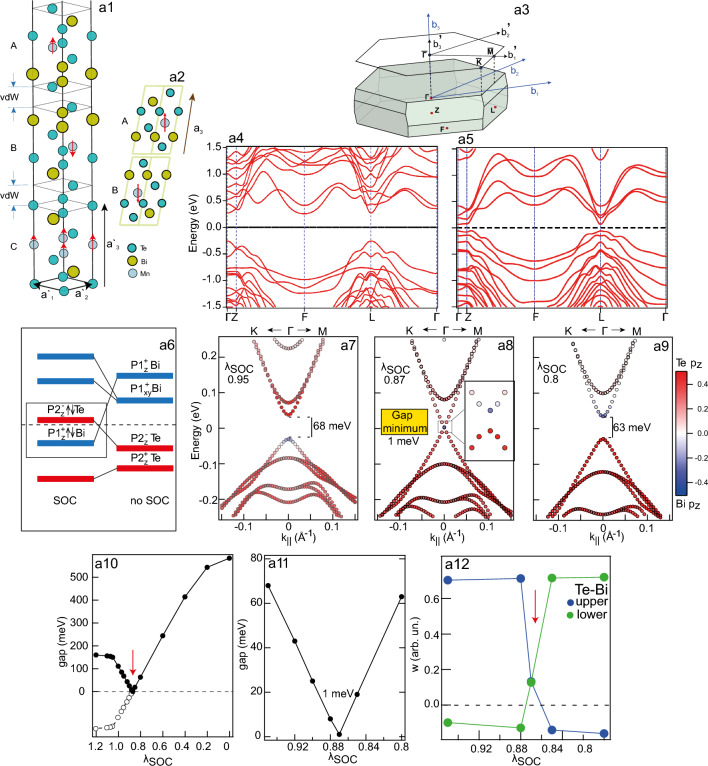
(**a1**) non-primitive unit cell (ABC stacking of SLs separated by vdW intervals) and (**a2**) primitive unit cell (AB stacking) of MnBi$_2$Te$_4$ with AFM ordering as well as their Brillouin zones (**a3**). Electronic structure corresponding to the primitive cell of MnBi$_2$Te$_4$ without (**a4**) and with (**a5**) SOC. (**a6**) Schematic diagram of the Bi $p1_z^+$ and Te $p2_z^-$ states inversion at the $\Gamma$ point for the $``on{\text{''}}$ and $``off{\text{''}}$ spin-orbit coupling in the bulk of the crystal (taken from ref.^31^). (**a7**–**a9**) The calculated band dispersions of non-primitive unit cell, showing changes in the bulk electronic structure near the $\Gamma$ point close to the region of the formed band gap minimum, which occur when $\lambda _\text {SOC}$ varies for all atoms in the crystal during the bulk TPT. (**a10**) Dependence of the band gap size on the relative change of $\lambda _\text {SOC}$ (filled black dots). Open dots show the change in the sign of the gap (see text). (**a11**) The region near the TPT (minimum of the band gap value) in the gap dependence. (**a12**) The changes in the difference of the Te $p_z$ and Bi $p_z$ contributions, taking into account the change in the sign of the difference, both for states near the top and bottom of the band gap. Here, too, the TPT point is shown by a vertical arrow.

Let us first analyze the possibility and conditions for implementation of the TPT which takes place in the MnBi$_2$Te$_4$ upon modulation of the SOC strength relative to normal value $\lambda _\text {SOC}$ (taken as 1) in DFT calculations of an infinite crystal. Fig. 1 shows primitive unit cell (a2) of MnBi$_2$Te$_4$ with AFM ordering as well as its Brillouin zone (a3) and the corresponding band structure with (a4) and without (a5) SOC. In order to directly compare the bulk electronic structure with the surface electronic structure we used a non-primitive unit cell (a1) consisting 6 SLs (taking into account ABC stacking of SLs and AFM ordering). Fig. 1a6 is a schematic presentation of the band inversion between the Bi $p1_z^+$ and Te $p2_z^-$ states (here ± are the parity indices), which corresponds to the transition from topological to trivial state when $\lambda _\text {SOC}$ changes from 1 to 0. The TPT supposedly occurs at a certain SOC strength $\lambda _\text {SOC}^0$ between 1 and 0 values where the band inversion takes place at the very edges of the bulk band gap. This is confirmed by analyzing DFT calculations of the bulk MnBi$_2$Te$_4$ crystal for different $\lambda _\text {SOC}$ values in this region and slightly higher than normal value. Some of the resulting bulk band dispersions near the $\Gamma$ point in the band gap region are shown in Fig. 1a7–a9 where the red color shows the Te $p_z$ contribution preponderance and the blue color shows the states with dominant the Bi $p_z$ contribution (the colors match those in the Fig. 1a6). The full set of these dispersions covering greater $\lambda _\text {SOC}$ range (without Bi/Te $p_z$ contribution analysis) is presented in Fig. 1S Suppl. Inform. The dispersions presented in Fig. 1a7–a9 show that when passing through the TPT point (with changing the SOC strength), there is indeed an inversion of the contributions of Te $p_z$ and Bi $p_z$ states at the edges of the bulk band gap at the $\Gamma$ point. It is clearly seen that for the topological insulator state ($\lambda _\text {SOC}\approx 0.95$ and higher) the contributions of the Te $p_z$ (Bi $p_z$) states are predominant at the band edges of the CB (VB), then for the state of a trivial insulator ($\lambda _\text {SOC}\approx 0.8$ and below) these contributions are inverted.

Figure 1a10 shows the bulk band gap values plotted for all values of $\lambda _\text {SOC}$ within the entire analyzed $\lambda _\text {SOC}$ range. According to this plot, with a decrease in $\lambda _\text {SOC}$ from the value slightly higher than in MnBi$_2$Te$_4$, the band gap value first decreases, and at $\lambda _\text {SOC}$ of about 0.87 it reaches a minimum value (1 meV) and with a further decrease in $\lambda _\text {SOC}$, the band gap value begins to increase again. Vertical arrow highlights the band gap minimum and its region is plotted in more details in Fig. 1a11. Finally, Fig. 1a12 quantifies the differences between the Te and Bi orbital contributions at the $\Gamma$ point for the valence and conduction bands at different $\lambda _\text {SOC}$, showing the abrupt interchange of these contributions when the band gap minimum is passed. According to the diagram in Fig. 1a6 the TPT point corresponds to the inversion of the Bi $p1_z^+$ and Te $p2_z^-$ states at the edges of the bulk band gap (and the corresponding inversion of the sign of the gap), therefore, this inversion is denoted in Fig. 1a10 by the filled and open symbols. So, we can state that TPT takes place in the (bulk) band gap minimum region corresponding to the inversion of the Bi $p1_z^+$ and Te $p2_z^-$ states. It should be noted that the presented band gap dependence is similar to those reported in literature^12–14^ which were obtained by different methods for other intrinsic AFM TIs, for example, $\text {Mn}_{2} \text {Bi}_{2} \text {Te}_{5}$ or $\text {Mn}_{2} \text {Bi}_{6} \text {Te}_{11}$ to analyze the possibility of observing the dynamic axion effect in these materials.

Thus, our presentation demonstrates that the gap actually changes its sign when passing through zero, which means that the TPT from the topological to the trivial state is realized in the system. We show that by changing $\lambda _\text {SOC}$ in the bulk of MnBi$_2$Te$_4$, it is indeed possible to achieve the conditions necessary for the bulk TPT from the topological to the trivial state with the inversion of the Bi $p1_z^+$ and Te $p2_z^-$ states at the bulk band gap edges, which can be represented as a change in the sign of the bulk gap at the transition point. As previously noted, this is precisely one of the most important conditions necessary for the realization of the axion-like state in AFM TI (the system should be in a state close to the TPT with minimal value of the gap^10–15^).

Now the question arises of how $\lambda _\text {SOC}$ variation will affect the change in the structure of the TSSs (as Dirac fermions localized on the surface, i.e., in the region of the spatial $\theta$-boundary or on the surface of AFM TI) in calculations for a slab with a surface.

### Modification of the surface electronic structure upon topological phase transition

**Figure 2:**
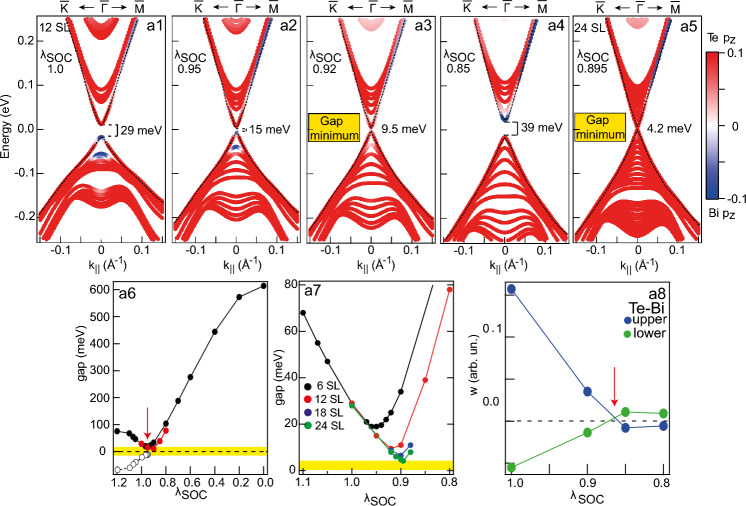
(**a1**–**a4**) Calculated electronic structure of the TSSs and the nearest VB and CB states for different $\lambda _\text {SOC}$ values for a slab with a thickness of 12 SLs. (**a5**) The similar dispersions calculated at the point of the Dirac gap minimum for a slab thickness of 24 SLs. TSSs in panels (**a1**–**a5**) are marked by dotted lines. (**a6**) Dependence of the Dirac gap size on the relative change of $\lambda _\text {SOC}$ for a slab thicknesses of 6 and 12 SLs (filled black and red dots). Open dots show the change in the sign of the gap (see text). (**a7**) The region near the TPT (minimum of the Dirac gap value) in the gap dependence for a slab with a thickness of 6, 12, 18 and 24 SLs (black, red, blue and green symbols, respectively). (**a8**) The changes in the difference in the (Te $p_z$ – Bi $p_z$) contributions, taking into account the change in the sign of the difference, both for states near the top and bottom of the Dirac gap for a slab with the thickness of 12 SLs. Here, too, the TPT point is shown by a vertical arrow.

Analysis of $\lambda _\text {SOC}$ dependence of the TSS band structure leads to similar conclusions regarding the nonzero gap minimum existence which were drawn earlier for the bulk band structure, however, in this case it takes place for the Dirac gap in the structure of the TSSs. The Dirac gap also decreases with a decrease in $\lambda _\text {SOC}$ value and reaches a minimum when $\lambda _\text {SOC}$ decreases to $\lambda _\text {SOC}^0$. With a further decrease in $\lambda _\text {SOC}$, a reverse increase in the energy gap begins to occur. Figure 2a1–a4 show the band structure of the TSSs and the nearest bulk bands of 12 SL MnBi$_2$Te$_4$ slab at some values of $\lambda _\text {SOC}$ including the normal value for MnBi$_2$Te$_4$ ($\lambda _\text {SOC}=1$), the point, which corresponds to the Dirac gap minimum, and two values to the left and right of the minimum. The color map in panels (a1–a4) shows the Bi/Te $p_z$ contribution predominance where it is evident that both the gap in the TSSs at the DP (Dirac gap) and the band gap are inverting with respect to the Bi/Te contributions after passing the band gap minimum point. At this point the TSSs transform into bulk states with inversion of the Bi $p1_z^+$ and Te $p2_z^-$ state contributions at the edges of the gaps, which indicates the corresponding change of the bulk gap sign under the transition from the topological to the trivial state. The minimum point itself reflects a pattern of the Bi/Te contributions, which is difficult to attribute to either a trivial or a topological insulator and should reflect the state nearby the TPT point between these two phases.

From the presented results, we can conclude that the TPT, which occurs with a decrease in $\lambda _\text {SOC}$, also manifests itself in the modulation of the energy gap at the DP in the TSSs, which is followed by the formation of the nonzero Dirac gap minimum (at $\lambda _\text {SOC}$ of about 0.9). It takes place with the corresponding changes in the contributions of the Te $p_z$ and Bi $p_z$ states at the edges of the Dirac gap (because the TSSs in MnBi$_2$Te$_4$ are also formed by the Bi $p1_z^+$ and Te $p2_z^-$ states, similarly as in $\mathrm{Bi}_{2}\mathrm{Te}_{3}$^30,44^), that can be a manifestation of this TPT in the TSSs structure.

It is also worth noting that the Dirac gap magnitude may be affected by an interaction between the slab surfaces (so-called finite-size effect, see refs.^12,28,45^), but it is widely accepted that these effects usually manifest themselves for thin slabs (6 SLs or less), hence they are usually disregarded for rather thick slabs (10 SLs and more). Our results, however, imply that these effects are rather pervasive since the TSS band gap minimum and corresponding $\lambda _\text {SOC}$ value depend significantly on the slab thickness. Figure 2a6,a7 demonstrates the TSS band gap dependence on the $\lambda _\text {SOC}$ value for 6 SL (black), 12 SL (red), 18 SL (blue) and 24 SL (green) slabs. The TPT $\lambda _\text {SOC}$ region is shown in more details in Fig. 2a7, which shows the band gap minimum value of 19 meV for a 6 SL slab, 9.5 meV for a 12 SL slab, 6.5 meV for a 18 SL slab and 4.2 meV for a 24 SL. The complete sets of calculated band structures for 6, 12, 18, 24 SL slabs with $\lambda _\text {SOC}$ variation are shown in Figs. 2S–5S of Suppl. Inf., respectively. Figure 2a8 quantifies the difference between the Te and Bi orbital contributions at the $\Gamma$ point for states near the top and bottom of the Dirac gap for a slab with the thickness of 12 SLs showing the band inversion when the gap minimum is passed.

**Figure 3:**
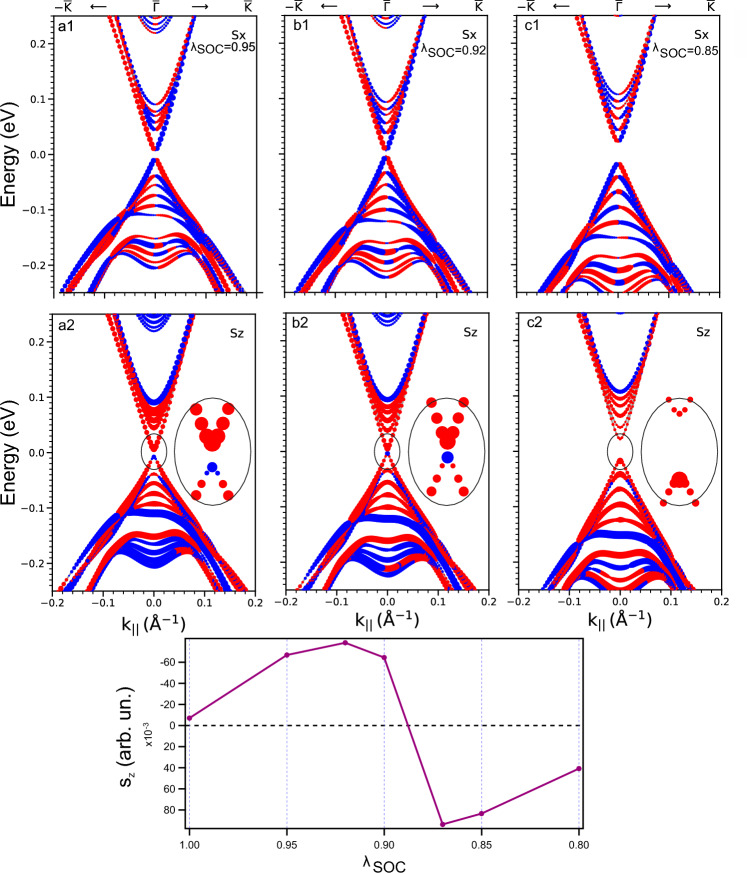
Modification of the in-plane (**a1**, **b1**,** c1**) and out-of-plane (**a2**, **b2**, **c2**) spin structure of the TSSs calculated for MnBi$_2$Te$_4$ (slab with thickness of 12 SLs) in the region of the $\Gamma$-point for the states of the upper and lower parts of the Dirac cone (red and blue dots), with a change in the SOC strength in the region of the TPT for the cases of above the transition point (**a1**,** a2**) when $\lambda _\text {SOC}= 0.95$; at the point of the TPT (**b1**, **b2**) when $\lambda _\text {SOC}= 0.92$ and below the TPT (**c1**, **c2**) when $\lambda _\text {SOC}= 0.85$. Insets in lower panels show more detailed presentation of the out-of-plane s$_z$ spin structure close to the region of the DP. (**d**) The change in the magnitude of the out-of-plane (s_z_) component of the spin polarisation of the Dirac cone states (averaged over two surface SLs) with a change in SOC strength.

Nevertheless, the thickness issue relates only to the TSS gap value since no other substantial changes to the band structure are observed when the slab thickness is increased. Figure 2a5, for comparison, shows the similar dispersion and the Bi/Te $p_z$ contribution calculated at the point of the band gap minimum for a slab thickness of 24 SL.

This allows the suggestion that the nonzero minimum gap values cannot be explained by the observed finite-size effects alone and their origins are of physical nature. This claim is also supported by similar observations of the TSS band structure in the non-magnetic $\text {Bi}_2 \text {Te}_3$ TI where a slab of 12 quintuple layer thickness exhibits the TSS gap value of $2 \times 10^{-2}$ meV.

In summary, the presented calculations for the slab show that as $\lambda _\text {SOC}$ decreases starting from the TI-state value, the Dirac gap also decreases, and at certain values the gap reaches its minimum corresponding to the TPT from the topological to the trivial state. At this point, the reinversion of the contributions of the Te $p_z$ and Bi $p_z$-derived states at the edges of the Dirac gap occurs, which corresponds to a change in the sign of the Dirac gap. In this case, the transition from the topological to the trivial state actually occurs with a jump through nonzero gap value. Thus, in the system with the surface, the condition necessary for the realization of the axion-like state (minimal gap value with inversion of the gap sign^10–15^) is also formed, only in this case this condition is realized for the TSSs.

### Modification of the spin structure upon topological phase transition

Figure 3 visualizes the influence of the TPT on the spin structure showing the band structure of the TSSs of a 12 SL slab with the in-plane $s_x$ projections in Figure 3a1,b1,c1 and the out-of-plane $s_z$ projections in Figure 3a2,b2,c2, while $\lambda _\text {SOC}$ is varied in the TPT neighborhood. Figure (a1, a2) correspond to the topological phase ($\lambda _\text {SOC}= 0.95 > \lambda _\text {SOC}^0$), Figs. (b1, b2) correspond to the TPT point ($\lambda _\text {SOC}= 0.92 \approx \lambda _\text {SOC}^0$) and Figs. (c1, c2) correspond to the trivial phase ($\lambda _\text {SOC}= 0.85 < \lambda _\text {SOC}^0$). It can be seen that in all cases the in-plane $s_x$ projections depicted in Fig. 3a1,b1,c1 demonstrate the spin-momentum locking typical for TIs. The analysis of the out-of-plane $s_{z}$ spin structure in the DP region for given values in the SOC strength, shown in Fig.  3a2,b2,c2 and corresponding insets demonstrates the fact that, indeed, for $\lambda _\text {SOC}$ above the TPT point (for the topological state) and nearby there is an out-of-plane spin polarization inversion for the states at the $\Gamma$ point. At the same time, for $\lambda _\text {SOC}$ below the TPT, i.e., in the trivial state, such an inversion is not observed. Thus, in the MnBi$_2$Te$_4$, when passing through the TPT point, a change in the sign of $s_{z}$ is actually observed, which is an another necessary condition for observing an axion-like state^10–15^. One can also notice that the magnitude of the out-of-plane ($s_z$) component of the spin polarisation of the bottom Dirac cone states (averaged over two surface SLs) with a change in SOC strength has a maximum at the TPT point ($\lambda _\text {SOC}= 0.92$), see Fig. 3d. It can be considered as some indirect confirmation of the formation of an axion-like state, conditioning the intercoupling between magnetism and SOC at the boundary between the topological and trivial phase in the TPT region corresponding to the region of the minimal (non-zero) Dirac gap.

### Modification of the topological surface states localization upon topological phase transition

**Figure 4:**
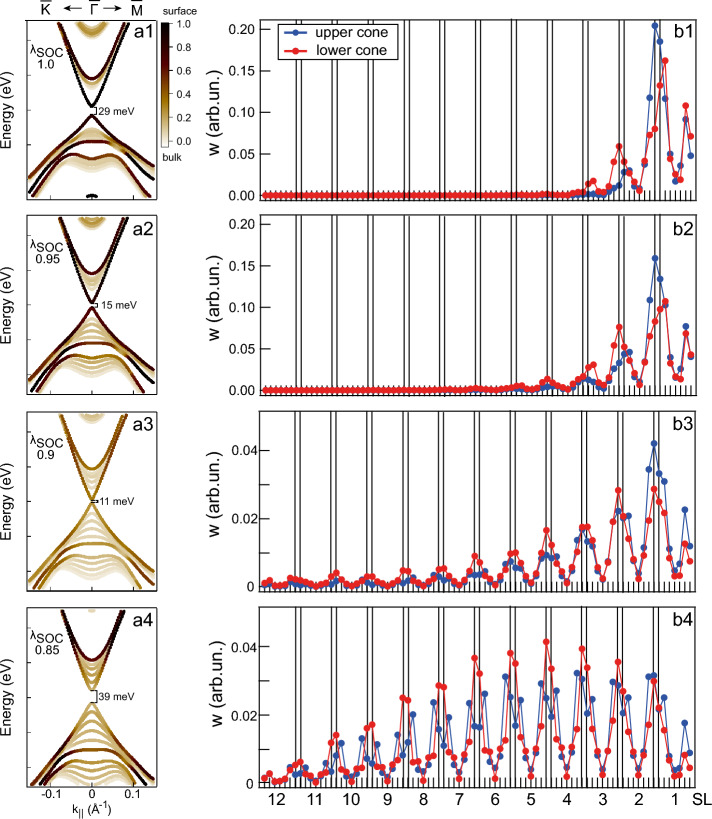
The band structures for $\lambda _\text {SOC}$ values corresponding the cases above (**a1**,**a2**), at (**a3**), and below (**a4**) the TPT point together with the demonstration of the changes in the localization of the states that form the TSSs (for a slab with thickness of 12 SLs). The brown dots and their sizes correspond to the states localized in the region of the first two surface SLs. Light brown colors correspond rather to the bulk-like localization. (**b1**–**b4**) Redistribution of TSSs density for the lower and upper parts of the Dirac cone (red and blue points) with modulation of $\lambda _\text {SOC}$ in the 12 SL slab with transition between surface- and bulk-like localization under the TPT.

Next, we will consider in detail changes in the localization of the states, which are considered as TSSs under normal conditions ($\lambda _\text {SOC}=1$), as well as with variations in $\lambda _\text {SOC}$ providing the transition through the TPT point from the topological phase to the trivial one. Since the atomic basis set is employed, this spatial distribution may be characterized by the distribution of its Bloch atomic weights which are defined for any slab atom *a* as $w_{a \textbf{k}} = \sum \limits _{m} |\langle \phi _{a m}| \psi _{\textbf{k}} \rangle |^2$ where $\phi _{a m}$ is the *m*-th basis orbital of the atom *a* where the sum runs over the whole basis set of the atom *a*.

The band diagrams in Fig. 4a1–a4 describe the coarse-grained spatial localization of all bands for cases of $\lambda _\text {SOC}= 1$ (a1), $\lambda _\text {SOC}> \lambda _\text {SOC}^0$ (a2), $\lambda _\text {SOC}\approx \lambda _\text {SOC}^0$ (a3) and $\lambda _\text {SOC}< \lambda _\text {SOC}^0$ (a4) calculated for a slab with thickness of 12 SLs. The first two diagrams which correspond to the topological phase show that the TSSs are mainly localized in the first two SLs which is typical for surface states. However, when the system is nearby the TPT point the states at the band gap edges acquires a rather noticeable bulk contribution. In the trivial phase, these states have apparent bulk localization.

Figure 4b1–b4 present a more detailed picture of $\psi _{\textbf{k}}$ spatial localization and contain the result of $\textbf{k}$-averaging of $w_{a \textbf{k}}$ values over three $\textbf{k}$-points closest to the $\Gamma$ point which were selected from the band diagrams in Fig. 4a1–a4. In the trivial phase below the TPT point (panel b4) these states have an overall bulk distribution with a slight preference for the first half of the slab. Increase of $\lambda _\text {SOC}$ value pushes these states towards the slab surface where they eventually transform into the TSSs when the TPT point is passed (panels b2, b3); as $\lambda _\text {SOC}$ becomes greater, the more tight becomes the surface localization (predominantly in the first two SLs for $\lambda _\text {SOC}= 1$, see panel b1). This analysis confirms that the TPT is not accompanied by a visible jump change in the localization of the states at the band gap edges when the system crosses the TPT point, but is rather characterized by some smooth change in the localization of these states nearby $\lambda _\text {SOC}^0$ value.

### Possibility of implementing the TPT in real systems

**Figure 5:**
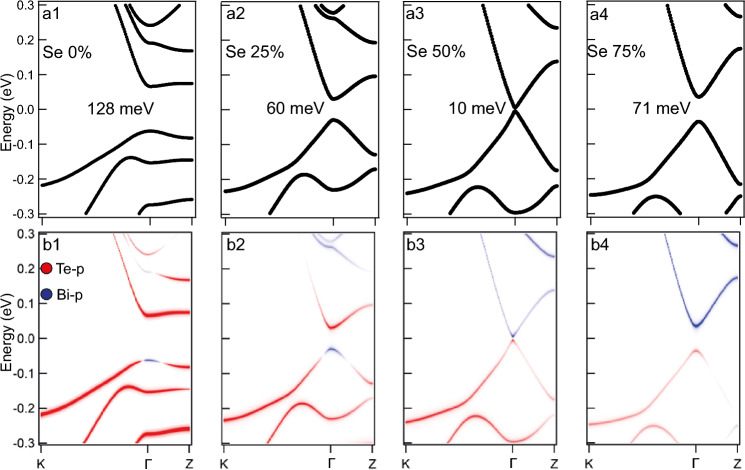
The calculated bulk band structure of $2 \times 2$ $\mathrm{MnBi}_{2}\mathrm{Te}_{4-x}\mathrm{Se}_{x}$ supercell along the K$\Gamma$Z path in the first Brillouin zone, the corresponding concentrations are *x* = 0 (**a1**, **b1**), *x* = 0.25 (**a2**, **b2**), *x* = 0.5 (**a3**,** b3**), *x* = 0.75 (**a4**, **b4**). The total spectral weight unfolded to $1 \times 1$ primitive cell is shown in black. Blue and red colours indicate regions of the Bi-*p* and Te-*p* orbital weight dominance, respectively.

**Figure 6:**
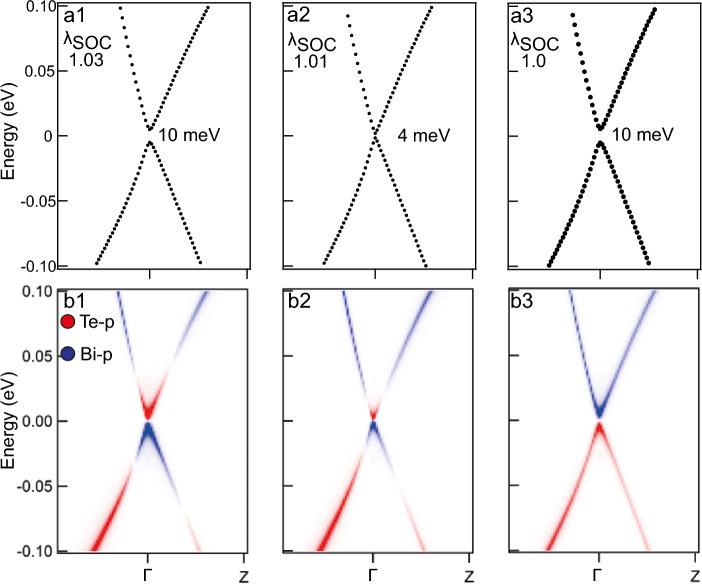
The calculated bulk band structure of $2 \times 2$ $\mathrm{MnBi}_{2}\mathrm{Te}_{2}\mathrm{Se}_{2}$ supercell along the K$\Gamma$Z path in the first Brillouin zone under a small $\lambda _\text {SOC}$ modulating relative to the spectra presented in Fig. 5a3, b3. The total spectral weight unfolded to $1 \times 1$ primitive cell is shown in black (**a1**–**a3**). Blue and red colours indicate regions of Bi-*p*$_z$ and Te-*p*$_z$ orbital weight dominance, respectively (**b1**–**b3**).

It should be noted that if we take the MnBi$_2$Te$_4$ as the basis, then in reality the above-mentioned variable calculated parameters related to the formation of the TPT (and the corresponding axion-like state) can be achieved by changing $\lambda _\text {SOC}$ by local replacement of Bi and Te atoms (with a large SOC value) by atoms with a smaller SOC value. In this work, we carried out the corresponding calculations with partial replacement of Te atoms by Se atoms. Figure 5a1–a4 show how the $K \Gamma Z$ bulk band structure of another AFM TI $\mathrm{MnBi}_{2}\mathrm{Te}_{4-x}\mathrm{Se}_{x}$ evolves with increasing concentration of Se atoms (only values of 0% ($x = 0$), 25% ($x = 1$), 50% ($x = 2$), 75% ($x = 3$) and 100% ($x = 4$) are accessible to the $2 \times 2$ supercell method). One may see that the increase of the Se concentration up to 50% leads to the bulk band gap decrease down to 10 meV, however, the gap increases if the Se concentration is further increased. This bulk band gap dependence is similar to the bulk band gap dependence on the $\lambda _\text {SOC}$ value in pristine MnBi$_2$Te$_4$ compound reflected in Fig. 1.

Figure 5b1–b4 show the evolution of the Te and Bi $p_z$ contributions into the states at the bulk band gap edges. It can be clearly seen that the band inversion disappears when the bulk band gap minimum point is passed towards larger Se concentrations. This indicates the possibility of the actual TPT in this system when $x \approx 2$ which is confirmed by $\lambda _\text {SOC}$ variation analysis for MnBi$_2$Te$_2$Se$_2$ system. In order to reach more real minimum of the band gap we have additionally varied slightly the SOC strength, as an analog of the effect that variation in the Se concentration has on the system. The calculations results presented in Fig. 6 show that even an 1% increase of $\lambda _\text {SOC}$ suffices to reach the minimum of the band gap with the corresponding band gap inversion. The real Se concentrations which are presumably sufficient to reach the TPT are estimated to be slightly lower than $50\%$ and even the MnBi$_2$Te$_2$Se$_2$ system itself may exhibit the TPT since the required $\lambda _\text {SOC}$ variation is rather small.

It can be seen that, when passing through the region near the minimum of the bandgap, a clear inversion of these states is observed at the edges of the gap. To find the exact position of the TPT point Fig. 6 shows changes in the gap size (Fig. 6a1–a3) and the corresponding changes in the Bi-$p_z$, Se-$p_z$ and Te-$p_z$ states contributions at the edges of the gap (Fig. 6b1–b3) for $\mathrm{MnBi}_{2}\mathrm{Te}_{2}\mathrm{Se}_{2}$ under $\lambda _\text {SOC}$ modulating (relative to the state shown in Fig. 5a3,b3). It can be seen that the minimum of the bandgap is reached at $\lambda _\text {SOC}=1.01$ (with respect to the initial SOC value taken as 1 for the system with $50\%$ of Se atoms). This corresponds to real concentrations of Se atoms slightly lower than $50\%$. It is very clearly seen that at the minimum point, the gap reaches a value of about 4 meV, and just between this point and the point related to the $50\%$ Se atom replacement, the contributions of the Bi-$p_z$, Se-$p_z$ and Te-$p_z$ states are inverted. Furthermore, it has been verified that the minimum energy in the $\mathrm{MnBi}_{2}\mathrm{Te}_{2}\mathrm{Se}_{2}$ calculations as well as for MnBi$_2$Te$_4$ corresponds to the AFM configuration, and the magnetic moments do not reveal deviations from the c-axis. Thus, the AFM TI with stoichiometry close to $\mathrm{MnBi}_{2}\mathrm{Te}_{2}\mathrm{Se}_{2}$ can be a real system, close in parameters to the TPT between the topological and trivial phase and, as a result, can be a practical platform for the implementation of the axion (axion-like state).

On the other hand, AFM TIs with MnBi$_2$Te$_4$ stoichiometry, showing the experimentally measured minimum gaps at the DP in the TSSs structure or gapless-like TSSs dispersions^34,38–40^, can also be considered as a possible platform for studying the axion-like state in Condensed Matter. It is assumed that the minimum value of the Dirac gap is achieved in these systems due to some little changes in parameters of the surface crystalline structure or the influence of various defects (in particular, the Mn/Bi substitution defects)^37,46,47^, which can lead to a significant decrease in the Dirac gap down to 3.5 meV^37^. According to Ref.^46^ the gap reducing and its probable collapse can be related to shifting of the system towards the TPT and corresponding change in the gap sign at certain defect concentration.

## Discussion

Thus, to summarize, the calculations show the following. In the case of an infinite MnBi$_2$Te$_4$ crystal (bulk calculations), the variation of $\lambda _\text {SOC}$ for its atoms is accompanied by the change in the size of the bulk bandgap, which reaches its minimum at the TPT point. At the same time, the contributions of the Bi $p1_z^+$ and Te $p2_z^-$ states are inverted at the edges of the bulk band gap. Presence of the surface in slab calculations leads to forming of the TSSs with the Dirac cone dispersion and the energy gap at the DP. In this case a change in $\lambda _\text {SOC}$ value provides the energy gap modulation in TSSs at the DP wherein the minimum (non-zero) value of the Dirac gap at the TPT point reaches 4.2 meV for the slab with the thickness of 24 SLs. Here the inversion of the states at the edges of the bulk band gap at the TPT point is transformed into the inversion of the TSSs at the edge of the Dirac gap that corresponds to the change in the sign of the gap.

In framework of the work devoted to the analysis of the possibility and efficiency of the implementation of the axion-like state in AFM TI using the massive Dirac Hamiltonian representation (the four-band Dirac model)^8,10–14^, it was shown that the largest fluctuations of the axion field can be achieved under the condition $\delta \theta =\frac{\delta m_5}{m_4}$, where $m_4$ and $m_5$ are determined by non-magnetic spin-orbit interactions (which do not violate time-reversal and spatial inversion) and magnetic (which violate time-reversal inversion) interactions, respectively. This means that for the system we are studying (MnBi$_2$Te$_4$), when $\lambda _\text {SOC}$ changes in the region of the TPT between topological and trivial phases (change in the axion term $\theta =\pi \rightarrow 0$), the conditions necessary for the formation of the axion-like state are just realized in the system:

- The system is characterized by the minimum gap at the DP in the TSS structure, the value of which is determined by the contribution of non-magnetic spin-orbit interactions ($m_4$ in the above expression). At the same time, at the TPT point, there is an inversion of the contributions of states with different parity at the edges of the Dirac gap, which indicates a change in the sign of the formed gap.
- At the TPT point, under the transition from the topological state of the system to the trivial one, an inversion of the TSSs spin structure is observed for the states of the Dirac cone at the $\Gamma$ point at the edges of the Dirac gap, which indicates the possibility of variation of the term $m_5$, determined by magnetic interactions.

At the same time, at the boundary between the topological and a trivial insulator (vacuum), i.e. at the surface of TI, a spatial topological $\theta$-transition also takes place, where the axion-like state should be spatially localized.

Wherein, in this case, all these conditions are achieved by reaching the TPT point with a change in $\lambda _\text {SOC}$ (without dynamics excitations), i.e. in this case, $\delta \theta$ depends on $\lambda _\text {SOC}$ and changes at the TPT point. In this case, the transition itself is characterized by the nonzero gap at the TPT point (4.2 meV or slightly less), i.e. the transition between the topological and trivial states of the system occurs with a “jump” through 0 in the range of possible gap values. We assume that it is the presence of the nonzero energy gap at the TPT point that is an indicator of the formation of the axion-like state, if it is considered as a result of the intercoupling between the spin-orbit and magnetic interactions at the TPT point, where the axion term changes, $\theta =\pi \rightarrow 0$ (see below).

**Figure 7:**
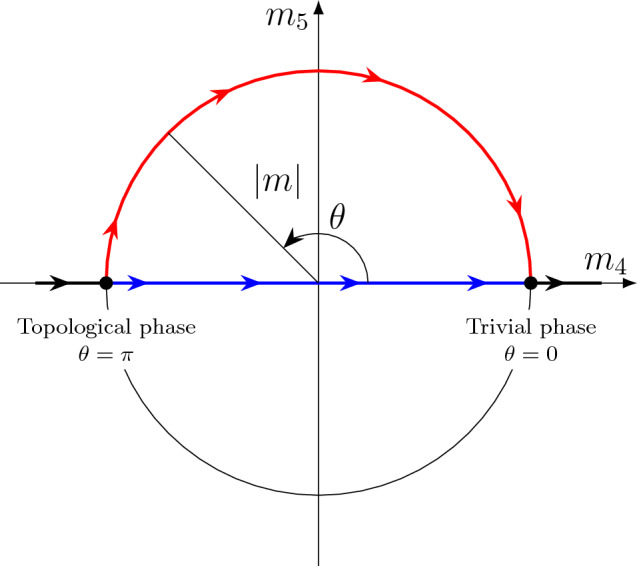
Complex plane schematic representation of relationship between the axion field $\theta$ and the nonmagnetic and magnetic orders $m_4$ and $m_5$ in the region of the TPT within the change $\theta =\pi \rightarrow 0$. $m_4$ corresponds to the non-magnetic order (mainly due to the spin-orbit coupling) that characterizes the topological phase transition, and $m_5$ corresponds to the antiferromagnetic order. Here, the topological state corresponds to the left point on the circle (with radius $\rho =|m|=\sqrt{m_4^2+m_5^2}$) on the axis $m_4$ ($\theta = \pi$), which is characterized by the negative Dirac gap ($m_4 < 0$). The trivial state corresponds to the right point on the circle ($\theta =0$), which is characterized by the positive Dirac gap sign ($m_4 > 0$). For AFM TI (like MnBi$_2$Te$_4$) the TPT takes place following the red arrows (without closing the Dirac gap). For non-magnetic TI with preserving the time-reversal symmetry the TPT takes place along the $m_4$ axis (at $m_5=0$) following the blue arrows without opening the gap.

This intercoupling can be visualized using the complex representation of the axion field by the variation of the contributions of non-magnetic (spin-orbit) and magnetic contributions (similar to ref.^22^). In this case, if we switch to the dependence of the magnitude of the energy gap at the DP, then $\theta$ can be represented as a phase of the complex mass term field as $m=\rho e^{i\theta }$ (see ref.^8,48^), as it is shown in Fig. 7. The possibility of a complex representation of the mass term under a variation of the axion field was already noted in the pioneering work^2^. With such a presentation the real and imaginary parts in this approach can be represented as $m_4$ and $m_5$, respectively, in the expression $m=m_4+{im}_5$. Here $m_4=\rho \cos {\theta }$ and $m_5=\rho \sin {\theta }$ with modulus $|m|= |\rho | = \sqrt{{m_4}^2+{m_5}^2}$. The term $m_4$ corresponds to the contribution of nonmagnetic spin-orbit interactions in framework of the massive Dirac Hamiltonian in the four-band Dirac model (see, for instance refs. ^8,10–14^), and the $m_5$ corresponds to the contribution of AFM interactions. In this representation, the topological state corresponds to the left point on the circle with radius $\rho =|m|=\sqrt{m_4^2+m_5^2}$ on the axis $m_4$ (the point $\theta =\pi$) in Fig. 7, which is characterized by the negative sign Dirac gap. The trivial state corresponds to the right point on the given circle (the point $\theta =0$), which is characterized by a positive sign Dirac gap.

This representation demonstrates the intercoupling between nonmagnetic spin-orbit and magnetic contributions under the transition $\theta =\pi \rightarrow 0$ and their possible mutual transformation at the TPT point. Similar representations of the change in $\theta$ at the transition between the topological and trivial phase without and with broken symmetry preserving parameters are presented in refs.^27,49^, where it is also shown that this transition in the region of $\theta$ change (between quantized values of $\pi$ and 0, when the symmetries are broken) occurs without gap closure at the TPT point. For the non-magnetic topological insulator, when the transition occurs exclusively along the horizontal axis ($m_4$ change), where the symmetry is preserved, $\theta$ jumps directly from $\pi$ to 0 at the TPT point. In this case, the gap at the TPT point is closed.

At the same time, this representation also allows to represent $\delta \theta$ as a function of $\frac{m_5}{m_4}$, which can be obtained as an approximation for small $\theta$ from the expression $\theta =\frac{\pi }{2}\left[ 1-\operatorname{sgn}\left( m_4\right) \right] -\arctan (\frac{m_5}{m_4})]$ at $m_5\ll m_4$^8^, for a system outside the point of the TPT. Here, the first term is 0 or $\pi$, which describe whether the system is topologically trivial or nontrivial. The second term describes possible deviations from the quantized value, really including not only under dynamics excitation. If we decrease the SOC strength (or $m_4$) for AFM MnBi$_2$Te$_4$ in the transition from a topological to a trivial system state in Fig. 7 (this is movement from left to right along the $m_4$ axis shown by red arrows), then at a certain moment (in the region of the TPT) the contributions of $m_4$ and $m_5$ become comparable (the left point on the circle in Fig. 7). In this case the condition for preserving the combined topological invariant for MnBi$_2$Te$_4$ (characterized by $\theta =\pi$) is violated. The value of $\theta =\pi$ starts to differ from the quantized values of $\theta =\pi$ and 0, and the conditions for the change in $\delta \theta$ appear, thereby affecting $m_4$ and $m_5$, within their relationship in accordance with this complex representation. As a result, an intercoupling appears between $m_4$ and $m_5$, and the system passes from the TI state ($\theta =\pi$) to the trivial insulator state ($\theta =0$) with a different parity and opposite sign of the energy gap at the $\Gamma$ point (already with non-inverted surface states at the edges of the gap). Wherein, this transition occurs by converting the electrical contribution ($m_4$) into the magnetic one ($m_5$) and again into $m_4$ within the change $\theta =\pi \rightarrow 0$, which can be described by the rotation of a vector with modulo $|m|$ in this representation (see red line in Fig. 7). It is the invariance of the modulus $|m|$ with changes in $\lambda _\text {SOC}$ in the region of the TPT that determines the invariance of the gap size during such a transition. In this case the gap size follows the expression $E_{gap}=\sqrt{m_4^2+m_5^2}$ at the point of the TPT, see also ref.^12^.

Some qualitative confirmation of the intercoupling between $m_4$ and $m_5$ (i.e., non-magnetic (spin-orbit) and magnetic interactions) at the TPT point can be provided by the change in the magnitude of the out-of-plane (*s*$_z$) component of the spin polarization of the bottom Dirac cone states with a change in the SOC strength, shown in  Fig. 3d, which has a maximum for the TSSs from the bottom Dirac cone directly at the TPT point ($\lambda _\text {SOC}=0.92$). The magnitude of the *s*$_z$ component in the TPT region turns out to be even larger in magnitude than for the original AFM TI MnBi$_2$Te$_4$ ($\lambda _\text {SOC}=1.0$), which can qualitatively testify in favor of the enhanced coupling between $m_4$ and $m_5$ in the TPT region (see Fig. 7). It is suggested that it is the enhanced coupling between $m_4$ and $m_5$ in the TPT region (when the $\theta$ value changes between $\pi$ and 0 at the boundary between the topological and trivial phase) that leads to the enhanced value of the out-of-plane polarization of the TSSs in the TPT region, which can be considered as some indirect confirmation of the formation of an axion-like state, conditioning the relationship between $m_4$ and $m_5$ at the boundary between the topological and trivial phase in the TPT region corresponding to the region of the minimal (non-zero) Dirac gap.

Meanwhile, it can be assumed that this intercoupling also determines a possibility of the topological ME effect in the region of the TPT and the formation of the axion-like state at the boundary between the topological and trivial phase (vacuum). In this case, the relationship between the gradient $\theta$ in the region of the TPT and the corresponding interconnected changes in the electric and magnetic fields can be represented by the Gauss law with the introduced axion term $\nabla \cdot \textbf{E} = \rho - \frac{\alpha }{4\pi ^2}\nabla \theta \cdot \textbf{B}$^2,8,50^. It means that the change $\theta =\pi \rightarrow 0$ (or the gradient of $\theta$) at the boundary between the topological and trivial medium just causes the corresponding change in electric and magnetic fields, i.e. the axion-like state, according to our assumption, can be represented as a quasiparticle due to the intercoupling between the electric (spin-orbit) and magnetic contributions to the generated ME response for a system with parameters close in parameters to the TPT between the topological and trivial phase.

Such a representation to some extent correlates with the effect of the axion instability^20–22^. Within the framework of this effect, when the electric field applied to the magnetic TI exceeds a certain level of $E_{crit}$ (in our case, at a certain value of the intra-atomic potential gradient, determined by $\lambda _\text {SOC}$, when the system in its parameters approaches the region of the TPT, i.e. when the value of $\theta$ begins to change (between 0 and $\pi$)), the electric field begins to be screened, leading to the inducing a local magnetic flux density in the region of the $\theta$-boundary due to ME effect ($B\left( \theta \right) =4\pi \mu M_\theta =\frac{\alpha }{\pi }\mu \theta E(\theta )$)^22^. In other words, the effect of axion instability can also be considered as the effect of a unique relationship between electric and magnetic fields in the region of the $\theta$ changing.

Interestingly, the estimated values of the nonzero minimum value of the energy gap at the DP at the TPT point (of about 4 meV) for the studied MnBi$_2$Te$_4$ and $\mathrm{MnBi}_{2}\mathrm{Te}_{4-x}\mathrm{Se}_{x}$ systems (which is supposed to match to twice the mass of the axion) correlate with the estimates of the axion mass in refs.^10,12,22^, where the magnitude of the axion mass for a system based on magnetic TI ($\mathrm{Bi}_2\mathrm{Se}_3+\mathrm{Fe}$) was estimated at the level of 1-3 meV. Here, it is worth noting that the $\mathrm{MnBi}_{2}\mathrm{Te}_{4-x}\mathrm{Se}_{x}$ system can serve as a good platform for studying the dynamic axion effect. The axion field fluctuations are maximized if a small fluctuation of some parameter of the system (e.g., $m_5$), induced by an external field, takes place for the system with the parameters close to the TPT, that can be established by considering the static characteristics of the system. We consider the value of our work precisely in the sense that we can propose not just an artificial change of the parameter $\lambda _{SOC}$, but a real system which according to results of calculations in its “$normal$” state ($\lambda _{SOC}=1$) already can be close to the phase transition (it is diagnosed on the basis of comparison of behavior of size of its bulk gap at variation of x parameter with artificial change of $\lambda _{SOC}$ in MnBi$_2$Te$_4$), and therefore there is sense to study its real response to application of external fields. A detailed description of the response for this system should be a subject of a separate theoretical and experimental investigations.

In the end we would like to note that such an approach to the problem of the axion-like state in Condensed Matter Physics as a mediator in the interaction between the topological and trivial phases (characterized by different parity of states) in the region of the TPT with localization of the axion-like state at the $\theta$-boundary (at the surface of TI) looks very promising and requires further detailed consideration.

## Conclusion

Comparative calculations of changes in the electronic and spin structure of MnBi$_2$Te$_4$ for an infinite crystal and a slab (analog of a crystal with a surface) have been carried out by the DFT method under variation of $\lambda _\text {SOC}$ in the region of the TPT between the topological and trivial states of the system. It is shown that at the TPT point both the bulk band gap (for an infinite crystal) and the gap open at the DP in the TSSs structure (for a slab of different thicknesses) reach their minimum with a decrease in $\lambda _\text {SOC}$, and then begin to increase again at the transition to a trivial state. Simultaneously, at the TPT point, the inversion of the contributions of the Bi $p1_z^+$ and Te $p2_z^-$ states with different parity at the edges of the Dirac gap occurs, thus testifying to the inversion of the sign of the gap, as well as the inversion of the spin *s*_z_out-of-plane polarization at the $\Gamma$ point for lower and upper parts of the Dirac cone. Wherein, for $\lambda _\text {SOC}$ higher than typical for TPT, the TSSs are localized near the surface, and when the system enters the trivial phase, the states that form the TSSs are already distributed over the entire bulk. The performed analysis showed that at the TPT point the system (AFM TI MnBi$_2$Te$_4$) has certain attributes necessary for the realization of the axion-like state localized at the $\theta$-boundary between the topological and trivial phases (or at the surface of TI). In this case, the transition from the topological to the trivial state of the system with a change in $\lambda _\text {SOC}$ takes place without closing the Dirac gap, i.e. with a “jump” through 0 and the formation of the nonzero gap during such a transition. We associate the nonzero Dirac gap with the formation of the axion-like state or the axion quasiparticle, which presumably couples nonmagnetic spin-orbit and magnetic contributions to the generated ME response at the $\theta$-boundary for a system with parameters close to the TPT between the topological and trivial phase. A complex representation of such an intercoupling is proposed for a change in $\lambda _\text {SOC}$ in the region of the TPT, where the axion term changes from $\pi$ to 0. At the TPT point, the system passes from the TI state ($\theta =\pi$) to the trivial insulating state ($\theta =0$) with a different parity and opposite sign of the gap at the $\Gamma$ point by mutual converting between electric and magnetic contributions within the change $\theta =\pi \rightarrow 0$, which determines the nonzero gap at the TPT. At the same time, it was shown that such a TPT with a bandgap minimum and inversion of the contributions of the Bi-$p_z$ and Te-$p_z$/Se-$p_z$ states at the edges of the gap can be practically implemented in the AFM TI with a stoichiometry close to that of $\mathrm{MnBi}_2\mathrm{Te}_2\mathrm{Se}_2$ with partial (about $50\%$) substitution of Te atoms (with a large SOC value) for Se atoms (with a smaller SOC value). This makes it possible to experimentally analyze the possibility of realizing the axion-like state and the corresponding topological ME effect in this AFM TI. Besides, such system could serve as a good platform for studying the dynamic axion effect, where the axion field fluctuations are maximised when a small external field is applied to the system which state is close to the TPT.

## Methods

Ab initio DFT calculations were carried out at the Resource Center $``Computer\,Center\,of\,SPbU{\text{''}}$ using the OpenMX code, which provides a fully relativistic DFT implementation with localized pseudoatomic orbitals^51–53^ and norm-conserving pseudopotentials^54^. The exchange-correlation energy in the PBE version of generalized gradient approximation was employed^55^. The accuracy of the real-space numerical integration was specified by the cutoff energy of 450 Ry, the total-energy convergence criterion was $1 \times 10^{-6}$ eV. The $\textbf{k}$-mesh for Brillouin zones were specified as follows: $5 \times 5 \times 5$ mesh for bulk calculations, $5 \times 5 \times 1$ for slab calculations with the vacuum layer of 12 Å. It was verified that further increasing the $\textbf{k}$-mesh density leads only to very weak changes in the electronic structure. As an unit cell we used the structure presented in the work^56^. The basis functions were taken as Bi8.0-s3p2d2f1, Te7.0-s3p2d2f1, Mn6.0-s3p2d1, Se7.0-s3p2d2f1 (the pseudopotential cutoff radius is followed by a basis set specification). The Mn 3*d* states were treated within the DFT + U approach^57^ within the Dudarev scheme^58^ where U parameter equals 5.4 eV^28^.

### Supplementary Information

Supplementary Information.

## Data Availability

The datasets used and/or analysed during the current study available from the corresponding author on reasonable request.
