# Supplementary material for: Topological phase transition in the antiferromagnetic topological insulator MnBi2Te4 from the point of view of axion-like state realization
Source: Sci Rep. 2023 Sep 28;13:16343. doi: 10.1038/s41598-023-42466-7 (PMC10539541; doi:10.1038/s41598-023-42466-7)
Supplement: Supplementary file 1 — Supplementary Information. [file 41598_2023_42466_MOESM1_ESM.doc]

**Supplementary Information**

**Topological phase transition in the antiferromagnetic topological insulator MnBi**_2_**Te**_4_ **from the point of view of axion-like state realization**

**A.M. Shikin**^1^,***, T.P. Estyunina**^1^**, A.V. Eryzhenkov**^1^**, N. Zaitsev**^2^**, and A.V. Tarasov**^1^

^1^ St. Petersburg State University, St. Petersburg, 198504 Russia

^2^ Institute of Molecule and Crystal Physics, Subdivision of the Ufa Federal Research Centre of the Russian Academy of Sciences, 450075, Ufa, Russia

*ashikin@inbox.ru

**Changes in the bulk electron structure with SOC modulation**


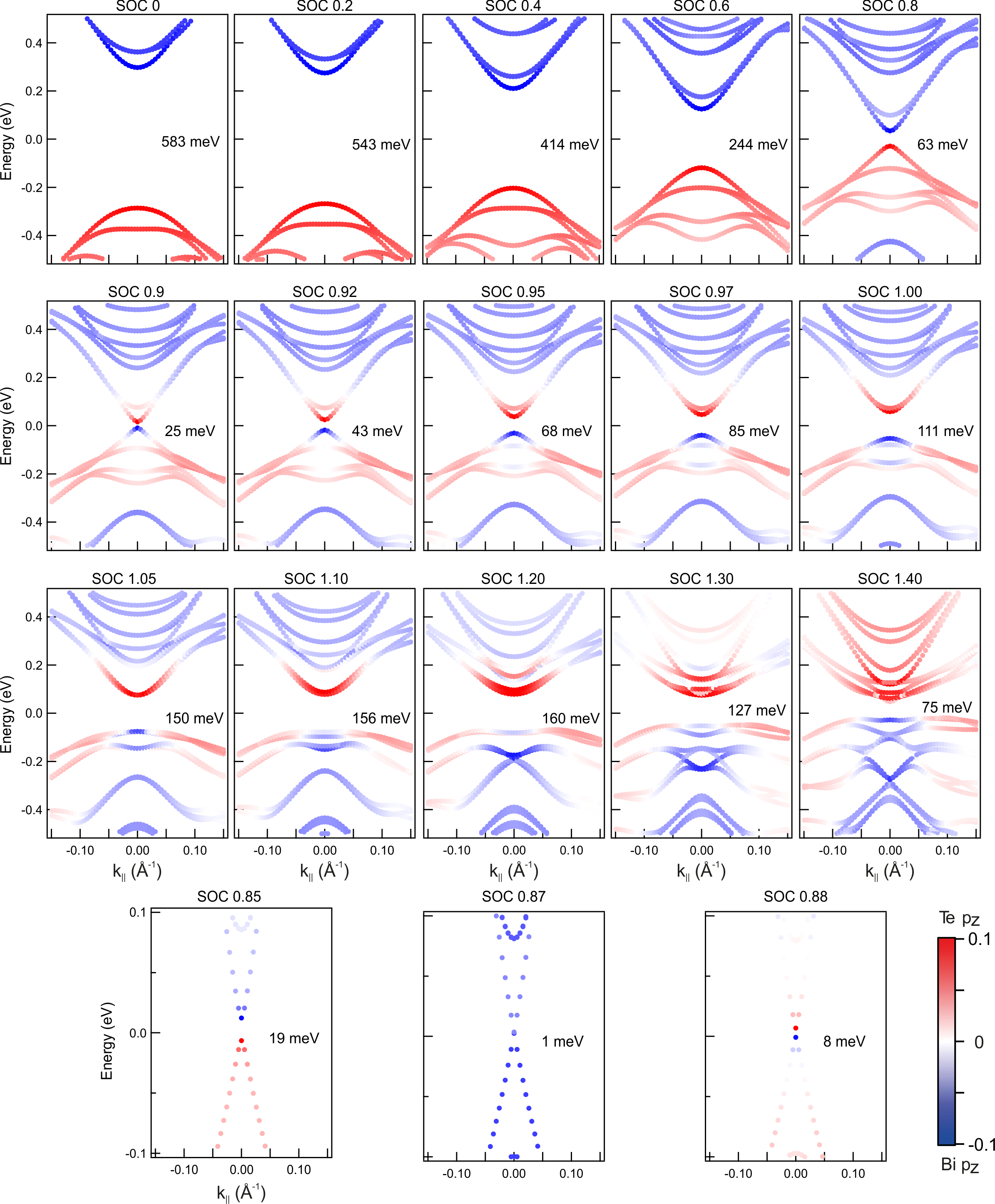


Fig. 1S. Changes of the bulk electronic structure for the states of the valence and conduction bands near the bulk band gap edges with variations of the SOC strength (relative to the value characteristic for the AFM TI MnBi_2_Te_4_, taken as 1) for all atoms of an infinite crystal. The values of SOC vary from 0 (SOC off) to 1.4. The minimum in the bulk band gap value (1 meV) at SOC=0.87 corresponds to the TPT region. The bottom row of inserts corresponds to the SOC strength region close the TPT point.

**Changes in the electronic structure of a 6SL slab with SOC modulation**

**
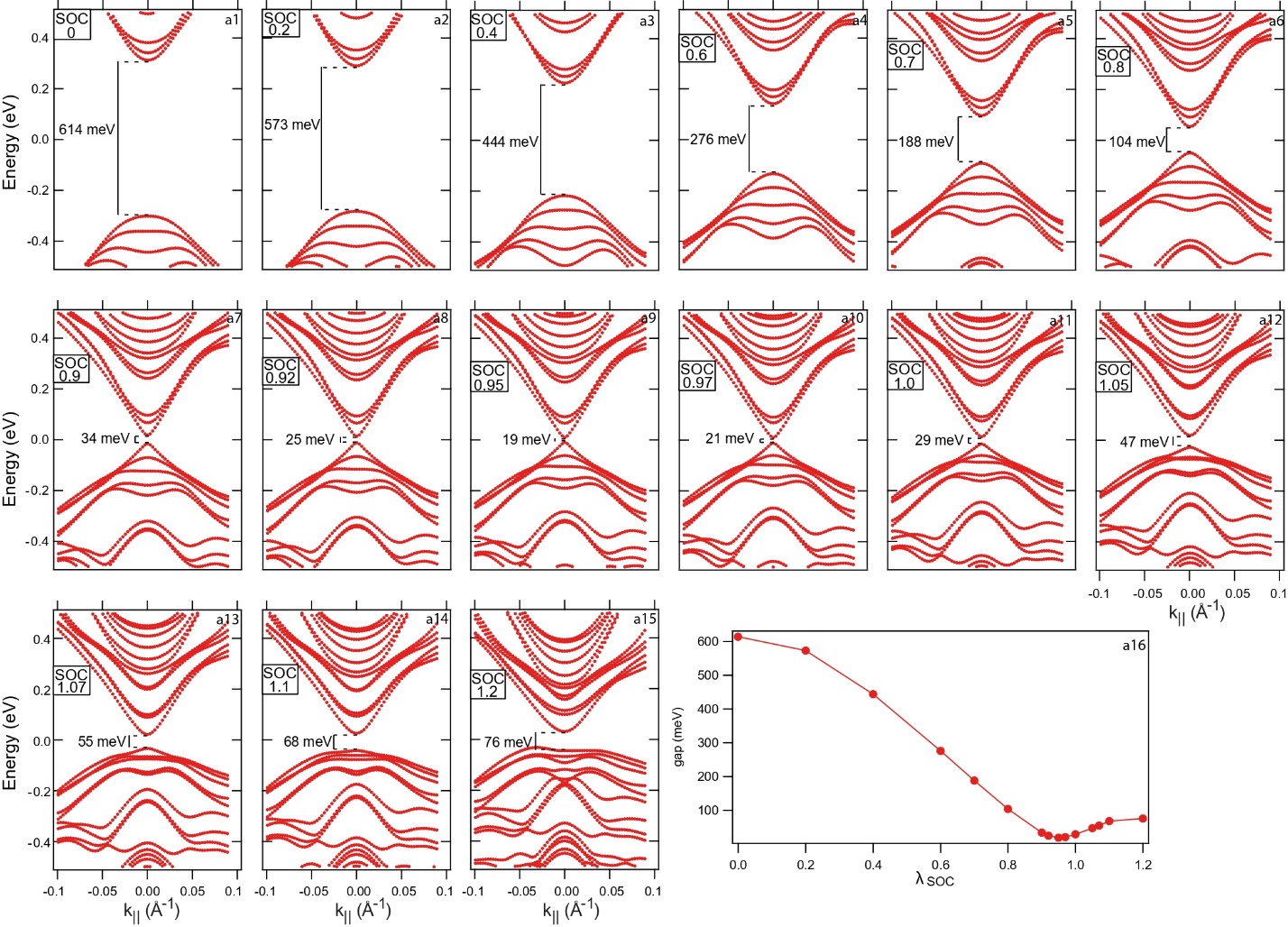
**

Fig. 2S. (а1-а15) - Changes of electronic structure of topological surface states and nearest states of valence and conduction bands for 6 SL thick slab corresponding to the variation of the SOC strength for all atoms in the slab (relative to the value typical for AFM TI MnBi2Te4, taken as a 1). Here, the SOC strength value is varied in the regions below the TPT point (0-0.92) and above the TPT point (0.97-1.2). The insets show the values of the energy gap in the TSS structure at the Dirac point. The minimum in the gap value (19 meV) at SOC=0.95 corresponds to the TPT region. Figure (a16) demonstrates changes in the Dirac gap value in the TSS with SOC modulation.

**Changes in the electronic structure of a 12 SL slab with SOC modulation**


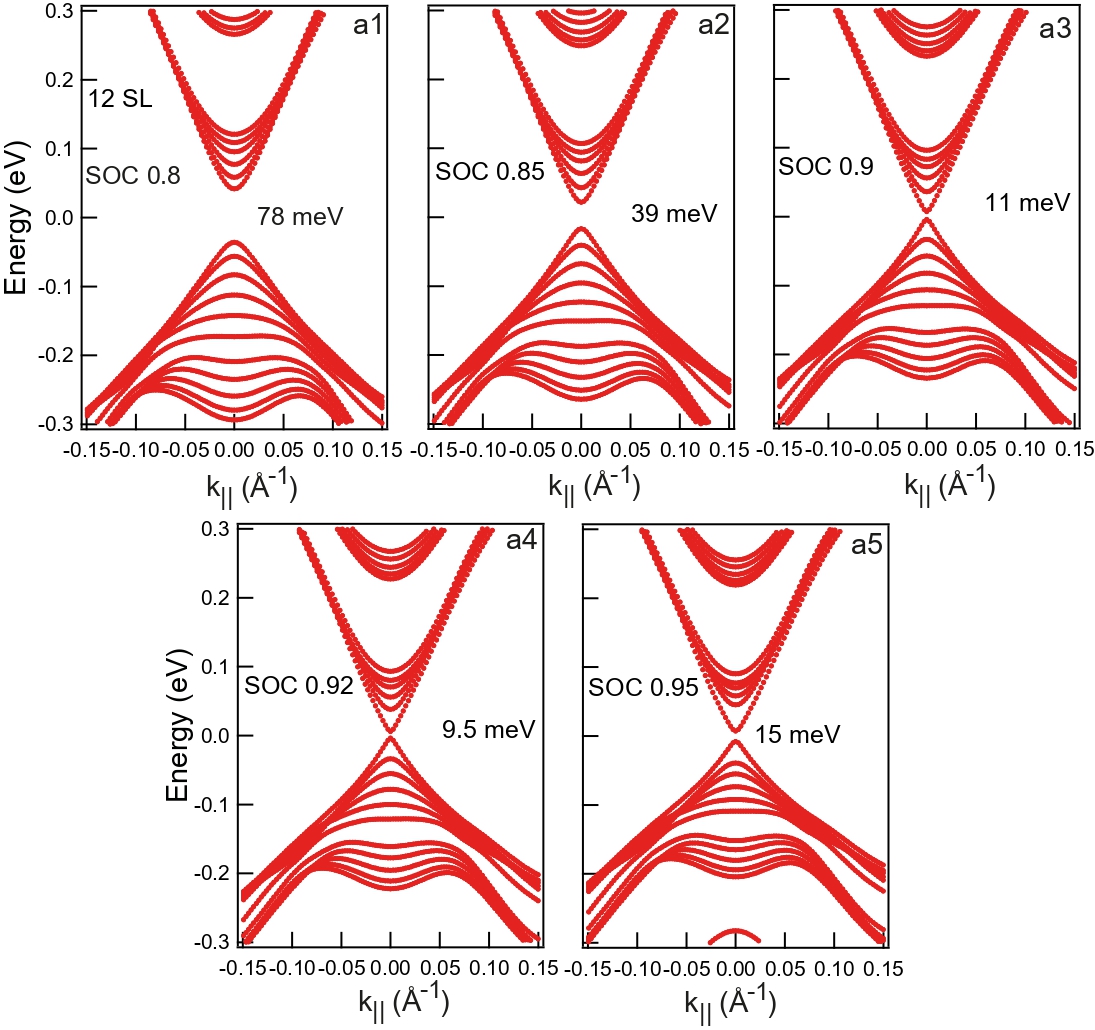


Fig. 3S. (а1-а5) - Changes of electronic structure of topological surface states and nearest states of valence and conduction bands for 12 SL thick slab corresponding to the variation of the SOC strength for all atoms in the slab (relative to the value typical for AFM TI MnBi2Te4, taken as a 1). Here, the SOC strength value is varied in the regions below the TPT point (0.8-0.85) and above the TPT point (0.92-0.95). In the insets the values of the energy gap in the TSS structure at the Dirac point are also shown. The minimum in the gap value (9.5 meV) at SOC=0.92 corresponds to the TPT region.

**Changes in the electronic structure of a 18 SL slab with SOC modulation**


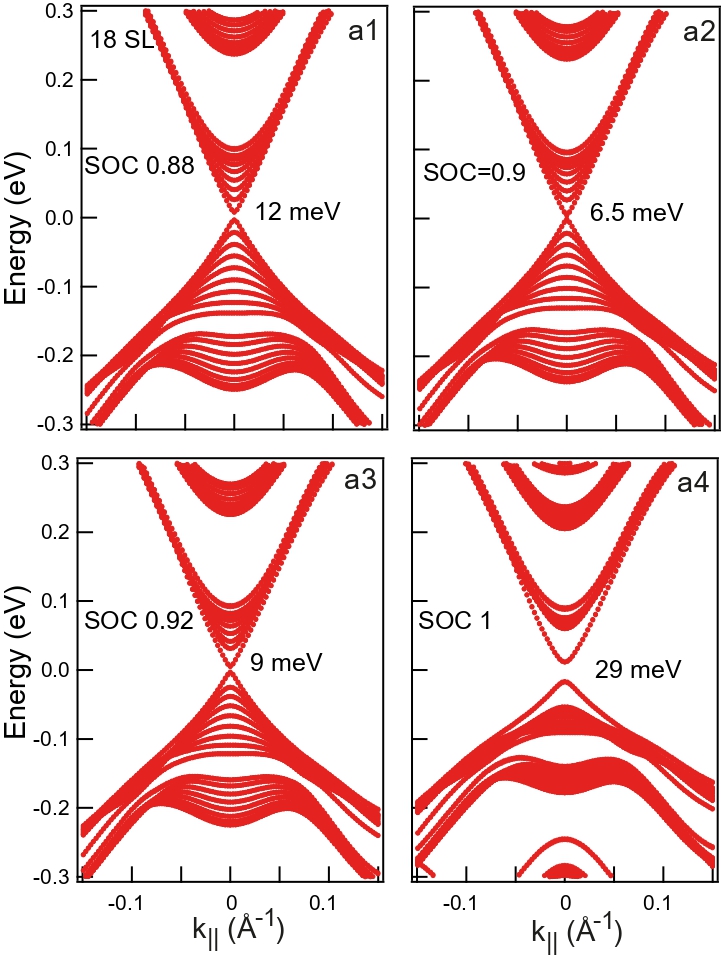


Fig. 4S. (а1-а4) - Changes of electronic structure of topological surface states and nearest states of valence and conduction bands for 18 SL thick slab corresponding to the variation of the SOC strength for all atoms in the slab (relative to the value typical for AFM TI MnBi2Te4, taken as a 1). Here, the SOC strength value is varied in the regions below the TPT point (0.88) and above the TPT point (0.92-1). The corresponding changes in the value of the energy gap in the TSS at the Dirac point are also shown here. The minimum in the gap value (6.5 meV) at the SOC strength of 0.9 corresponds to the TPT region.

**Changes in the electronic structure of a 24 SL slab with SOC modulation**


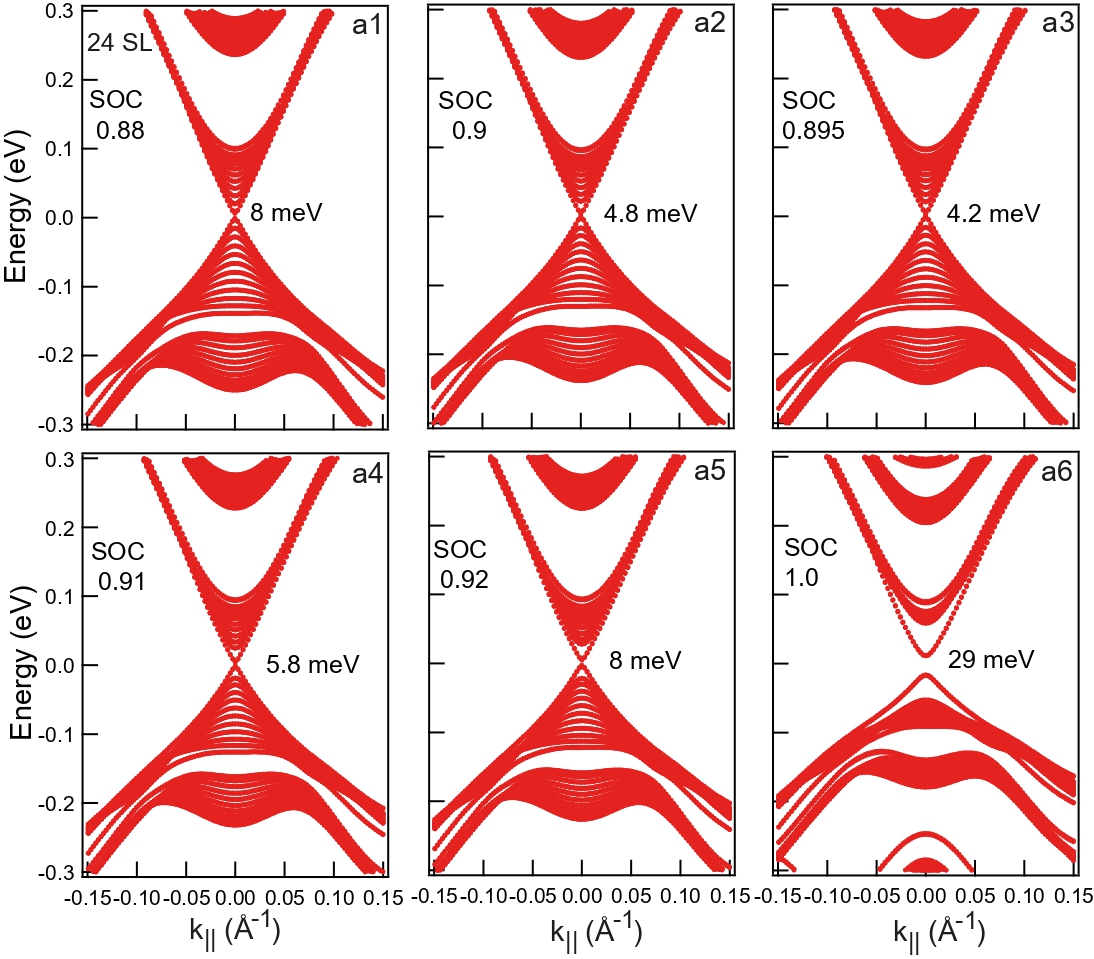


Fig. 5S. (а1-а6) - Changes of electronic structure of topological surface states and nearest states of valence and conduction bands for 24 SL thick slab corresponding to the variation of SOC strength for all atoms in the slab (relative to the value typical for AFM TI MnBi2Te4, taken as a 1). Here, the SOC strength value is varied in the regions below the TPT point (0.88) and above the TPT point (0.91-1). The corresponding changes in the value of the energy gap in the TSS at the Dirac point are also shown here. The minimum in the gap value (4.2 meV) at the SOC strength of 0.895 corresponds to the TPT region.

**Inversion of Te pz and Bi pz state contributions at the Dirac gap edges with SOC modulation at the topological phase transition**


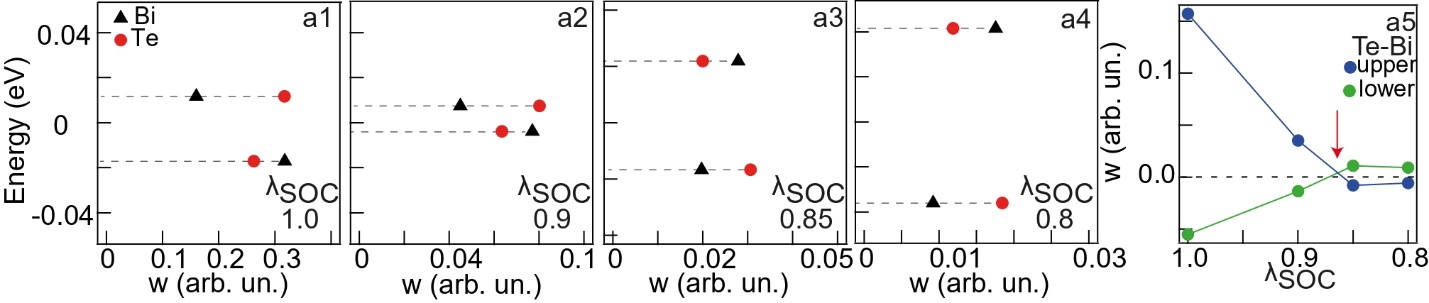


Fig. 6S. (а1-а4). Changes in the contributions of the Te pz and Bipz states at the edges of the Dirac gap under the variation of the SOC strength in the 12SL slab. (a5) - Resulting changes in the sign of the difference in the contributions of the (Te pz-Bi pz) of states at the edges of the Dirac gap with the SOC strength variation. The vertical red arrow corresponds to the TPT region.
